# Insurers' intervention, separation of two rights and firm's technology innovation

**DOI:** 10.3389/fpubh.2023.1181219

**Published:** 2023-04-12

**Authors:** Bin Xu, Fangjing Hao

**Affiliations:** ^1^Graduate School, The Seoul School of Integrated Sciences and Technologies, Seoul, Republic of Korea; ^2^School of Computer Science and Technology, Shandong Technology and Business University, Yantai, China

**Keywords:** insurance capital, insurance companies, technological innovation, separation of two rights, state-owned enterprises

## Abstract

This paper takes insurers' intervention as the entry point, and sets insurers' intervention, separation of two rights and firms' technological innovation in a specific context to study the transmission mechanism and economic consequences using panal model. The results show that there is a positive relationship between insurers' intervention and firm's technological innovation, and the degree of separation of two rights has a negative moderating effect on the relationship between insurers' intervention and technological innovation, and this effect is more obvious in the sample of state-owned enterprises. Therefore, the state should formulate relevant policies to guide the equity investment behavior of insurance companies so as to improve the operational efficiency of market resources.

## 1. Introduction

On August 16, 2019, People's Daily published “Insurance Funds Support the Real Economy and Technological Innovation through Stock Investment”, which puts forward new requirements on the concept of insurance companies in capital investment to reasonably allocate capital resources and focus on the technological innovation development of enterprises, thus improving the ability of financial services to the real economy. So how insurance funds entering the stock market will affect the technological innovation ability of enterprises is a topic worth studying.

Technological innovation, as a long-term non-productive activity of the firm, gives public companies an advantage over the general firm because of the large number of investors and thus the risk transfer. However, this advantage is likely to be weakened in the face of the agency problem of separation of two rights between management, as the pursuit of short-term profit goals and the rise of risk will make them pay less attention to corporate governance, thus reducing the technological innovation activities of the company (1). In general, when there are only individual investors, most of the investment agents will “vote with their feet”, being short-sighted and focused on short-term performance. However, when institutional investors join, the situation changes somewhat. Because institutional investors pay more attention to the long-term value of the company, especially the “hand voting” approach, they will participate in the internal governance of the company in order to pursue the long-term value of the company's development. In addition, institutional investors have some advantages, they have sufficient liquidity and abundant human resources to collect sufficient information to reduce the information asymmetry in R&D (2), so as to enhance the level of investment in R&D and thus improve the long-term value of the company. Although institutional investors have many advantages, different types of investors still play different roles in strengthening the internal management of firms, for example, only focused investors strengthen corporate governance, while temporary investors are counterproductive (3). And institutional investors do not necessarily strengthen corporate governance in 1990, and their relatively frequent transactions are not conducive to the long-term management of the firm. Bushee (4) further found that: when the shareholding of institutional investors is high, company managers are less likely to reduce R&D expenditures, thus increasing the technological innovation capacity of the company; at the same time, when the turnover rate is high and the market is dominated by impulsive investors, managers will reduce R&D investment, thus preventing the risk of profit downside. However, the impact of market sentiment may not reduce the company's financial stability and market share (5). So insurance companies, as important institutional investors, how do they affect technological innovation in the process of internal governance of the company after their participation in the company? Through what mechanisms when they act on technological innovation in the face of the different separation of two rights and agency problems of the company? This question is worth studying.

Therefore, this paper puts insurers' intervention, separation of two rights and technological innovation in one context and finally finds that the relationship between insurers' intervention and technological innovation is positive, and the degree of separation of two rights has a negative moderating effect on the relationship between insurers' intervention and technological innovation, and this effect is more pronounced in the SOE sample.

The contributions of this paper is as follows: Firstly, many papers have studied the relationship between institutional investors and enterprises from the perspective of institutional investors, and less from the perspective of insurance companies, but this paper enriches the study of the relationship between insurance companies' equity investments and capital markets. Third, the findings of this paper provide some empirical references for insurance companies to further plan their equity investment capital allocation to serve the development of the real economy.

## 2. Literature review, theoretical analysis and research hypothesis

Institutional investors have been major players in emerging capital markets and are important in terms of their influence on corporate governance, which can affect firms' investment decisions (6). As for the research on institutional investors and technological innovation, some scholars believe that institutional investors can promote technological innovation (7–9), mainly through the improvement of corporate governance (10), which is mainly reflected in the fact that institutional investors supervise and manage the company and thus improve the efficiency of the company's operations, and when the higher the shareholding of institutional investors, the less they reduce the company's R&D expenditure (4), which may be for the maintenance of established interests. As an important institutional investor, one of the important operations of insurance companies is information gathering and control measures to achieve efficient information management and risk management to minimize the cost of risk (11). The insurance companies have a strong data base and customer base, and they are gradually equipped with data mining capabilities in the process of self-competition in the insurance industry (12). And institutional investors have more positive behavioral motivations (13), which are crucial for promoting corporate governance, and are more likely to generate more original innovations in the capital market (14). And the increased confidence in the market is more helpful to the company's insurance business, contributing to the positive development of the market (15). In these idiosyncratic contexts, once an insurance company participates in the stock, it will first strengthen the internal governance and supervision of the company, and guide the company to make more accurate R&D investment decisions and improve the efficiency of capital allocation. At the same time, the higher the percentage of insurance participation, the more they will focus on the long-term development of the company, and will not easily withdraw the funds invested in R&D, which will guarantee the sustainability of R&D and increase the company's technological innovation.

However, investors are irrational, and institutional investors are no exception (16). Insurance companies may have a narrow view and thus can be driven by interest to influence managers to make short-term decisions that only increase short-term profits (17). Collusion with managers' interests can make the asymmetry of information disclosure more pronounced, especially in insurance companies where the contagion effect caused by information asymmetry is more severe (18). Asymmetric information can directly affect a company's investment decisions and have a direct impact on business performance. However, institutional investors tend to spend less on R&D when the company's operating performance is poor (4). Therefore, when the insurance company appears to collude with the interests of the company's management, it is likely to have information asymmetry, which generates excessive liability risk and thus affects the investment decisions of the company, and if the company's performance declines at this time, it is likely to reduce its R&D expenditures even if the company's shareholding ratio is high, otherwise it will affect the loss of greater interests. So what kind of influence do insurance companies have on corporate technology innovation? Therefore, the following competing hypotheses are proposed.

H1a: Insurers' intervention will promote the development of firm's technological innovation.
H1b: Insurers' intervention will inhibit the development of firm's technological innovation.

Compared with the mature capital market environment in Europe and the United States, the second type of agency problem of controlling shareholders manipulating the management of the company through the advantage of equity concentration is more serious in the special market environment and governance model in China. In the second type of agency problem, the ultimate controlling shareholders' control as well as cash flow rights of listed companies at the corporate governance level may deviate (19), hoping to gain a larger control and thus control the listed company with a very small cash flow right, indicating that the separation of two rights problem is more serious in Chinese listed companies. From the ultimate profit pursuit of shareholders, some scholars argue that the degree of separation of two rights plays a positive role in corporate technological innovation. From the risk diversification hypothesis, it is believed that the degree of separation of two rights helps major shareholders to diversify their risks and thus diversify their investments, which positively affects the R&D activities of enterprises on the basis of financial stability; and from the hollowing out theory under the pyramid structure, in the context of capital market innovation as a hot spot, major shareholders may passively increase R&D funds for technological innovation in order to meet the market hot spot. Therefore, it is believed that the separation of two rights will increase the technological innovation ability of enterprises (20). Moreover, although the rise of institutional investors will lead to the increase of equity concentration, they will effectively manage the agency problem (21), especially a small number of institutional investors own most of the company's shares, and they will strengthen supervision and management for their own interests and improve the efficiency of all aspects of the company's investment. Therefore, after the involvement of insurers, they will strengthen corporate governance through external supervision and other actions, especially paying high attention to the innovative behaviors related to the company's performance, thus improving the company's technological innovation capacity in general.

However, changes in the internal institutional environment of the company will affect the technological innovation capability of the company, and the technological innovation capability of the company will show an increase and then decrease during the change of the institutional environment from low to high, and the higher separation of two rights will also weaken the influence of the internal institutional environment on technological innovation (22), so the degree of separation of two rights may weaken the technological innovation capability (23). From the perspective of differences in the ownership structure of ultimate controllers, Chen et al. (24) argue that the degree of separation of two powers has a significant encroachment effect on the development of technological innovation and can produce significant distortions in the efficiency of corporate innovation. From the perspective of small and medium-sized listed companies, due to the scarcity of resources, shareholders generally engage in investments with high certainty and low risk, but major shareholders will engage in profit-making behaviors such as “tunneling”, which will invariably capture the company's profits, thus negatively affecting the development of technological innovation (25). After the insurer's acquisition, it may collude with the management of the listed company to seize the company's profit, which makes the company's internal governance low and its investment decision making ability decrease, and the level of technological innovation will not be improved. So the following hypothesis is proposed.

H2a: The degree of separation of two rights has a positive moderating effect on the relationship between insurers' intervention and technological innovation.
H2b: The degree of separation of two rights has a negative moderating effect on the relationship between insurers' intervention and technological innovation.

Compared with mature foreign markets, the most fundamental characteristic of Chinese listed companies is the state-owned enterprise background. The unique property rights nature of SOEs gives them a different role orientation. Most SOEs' decisions and actions are not aimed at purely economic interests, but rather at maximizing national interests, maintaining economic development, and social stability. As the largest shareholder, state-owned shareholders will form a certain conflict of interest and threat to other shareholders, which will create a certain information asymmetry problem and agency conflict. The major shareholders will engage in related transactions and appropriation of funds for their own interests, which will inevitably harm the interests of small and medium shareholders and further cause agency conflict, and the optimal allocation of resources cannot be better. The agency problem of separation of two rights in SOEs is more prominent than in private enterprises, and the opportunistic behavior of managers will have a greater impact on the behavior of institutional investors. In SOEs with more severe agency problems, the degree of separation of two rights plays a more significant effect on the relationship between insurers' intervention and technological innovation. Therefore, the following hypothesis is proposed.

H3: The moderating effect of the separation of two rights on insurers' intervention and technological innovation is more pronounced in SOEs relative to the private enterprise sample.

## 3. Study design

### 3.1. Sample selection and data sources

In order to avoid the impact of major accounting standard revisions, this paper selects the data of A-share listed companies from 2007 to 2018 as the sample for research and analysis, and follows the following principles: (1) financial listed companies are excluded; (2) companies marked ST or ^*^ST in the year are excluded; (3) some companies with missing data; (4) in order to exclude the influence of extreme values, all continuous variables are used at the 5% level. The financial data were obtained from CSMAR database and RESSET financial database, and cross-checked.

### 3.2. Model construction and variable definition

#### 3.2.1. Variable definition

(1) Technological innovation. Referring to Chang et al. (26)'s study, this paper uses the number of corporate patents as a proxy variable for corporate technological innovation. Technological innovation measured in this paper refers to the cumulative number of patents applied for, obtained, granted or accepted as of the end of the reporting period, and is logarithmically processed.

(2) Insurers' intervention. If the listed company has insurance company's participation, IP = 1, otherwise IP = 0; SP then indicates the percentage of insurance company's participation.

(3) Separation of two rights. Since agency conflict is mainly caused by the inconsistency of agent and principal's objectives, which leads to principal-agent problems and conflicts, the separation degree of two rights of control and ownership is selected as the agency variable of agency conflict in this paper. As shown in Table 1 is variable definition.

**Table 1:** Definition of variables.

**Variable**	**Variable description**
LnTI	Logarithm of the cumulative number of patents applied for, obtained, granted or accepted as of the end of the reporting period
IP	Whether the insurance company participates in the stock, if so, 1, otherwise, 0
SP	Insurance company's shareholding ratio
SPRT	The degree of separation of two rights, i.e., the difference between control and ownership
SOE	Nature of listed company, if state-owned,1, otherwise, 0
TQ	Tobin's Q = (market value of owner's equity + total book value of debt) / book value of total assets, i.e., the market value of the company as a proportion of the replacement cost of assets, if >1 indicates that the purchase of capital goods for the expansion of production is profitable, more investment is added; otherwise, the investment will be reduced
CFLOW	Free cash flow = (operating profit + depreciation and amortization)/total assets at the beginning of the year
SALES	Sales ratio = sales revenue / total assets at the beginning of the year
TOER	Change of hands ratio = Volume of stock traded during the year/Number of A shares circulating
LEV	Gearing ratio = Total liabilities at the end of the period/Total assets at the end of the period
BTM	Book-to-market ratio = Total assets at the end of the period/(market value of equity + market value of net debt)
ROA	Return on Assets = EBITDA / Total Assets at the end of the period
SIZE	Asset size, as the natural logarithm of total assets at the end of the period
Year	Annual dummy variables
Indu	Industry dummy variables

#### 3.2.2. Model construction

In order to verify the relationship between insurers' intervention and firm's technological innovation, this paper proposes to construct the following models: firstly, we test the relationship between insurers' intervention and firm's technological innovation through model (1) and model (3) to test hypothesis 1; next, we test the effect of the degree of separation of two rights on the relationship between insurers' intervention and technological innovation through model (2) and model (4) to test hypothesis 2 and hypothesis 3.


(1) $$\begin{array}{l} LnTI_{t}=\beta_{0}+\beta_{1}IP_{t}+controls+year+indu+\varepsilon \end{array}$$

$$\begin{array}{ll} LnTI_{t}= & \beta_{0}+\beta_{1}IP_{t}+\beta_{2}SPRT_{t}\times IP_{t}+controls+year+ \end{array}$$

(2) $$\begin{array}{l} indu+\varepsilon \end{array}$$

(3) $$\begin{array}{l} LnTI_{t}=\beta_{0}+\beta_{1}SP_{t}+controls+year+indu+\varepsilon \end{array}$$

$$\begin{array}{ll} LnTI_{t} & =\beta_{0}+\beta_{1}SP_{t}+\beta_{2}SPRT_{t}\times SP_{t}+controls+year+ \end{array}$$

(4) $$\begin{array}{l} indu+\varepsilon \end{array}$$

## 4. Analysis of empirical results

### 4.1. Descriptive statistics results

The results of descriptive statistics in Table 2 show that the standard error of enterprises in technological innovation is 5.352, which indicates that the heterogeneity of technological innovation varies very much among enterprises and the distribution of innovation results is very uneven; the standard deviation of the degree of separation of two rights is 6.7, which indicates that the heterogeneity of the difference between control and ownership of each company is relatively large; while the mean value of IP is 0.094 and the standard error of 0.292 indicates that the proportion of insurers' intervention in listed companies is still relatively large; the standard error of 0.003 for SP indicates that the variation in the proportion of insurers' intervention is not obvious.

**Table 2:** Descriptive statistics results.

**Variable**	**Mean**	**Std.Dev**.	**Min**	**Max**
LnTI	4.975	5.352	1.610	6.724
IP	0.094	0.292	0	1
SP	0.001	0.003	0	0.012
SPRT	4.2	6.7	0	20.4
TQ	2.418	1.701	0.445	6.689
CFLOW	0.022	0.012	0.005	0.049
SALES	0.236	0.427	−0.303	1.401
TOER	1.778	1.011	0.402	4.085
LEV	0.382	0.199	0.077	0.743
BTM	0.701	0.555	0.15	2.248
ROA	0.062	0.04	−0.005	0.148
SIZE	21.731	1.058	20.281	24.183
SOE	0.323	0.468	0	1
TAT	0.614	0.308	0.213	1.367

### 4.2. Main test results

Table 3 reports the test of the relationship between insurers' intervention and technological innovation with LnTI as the explanatory variable and IP and SP as the explanatory variables, where both columns (1) and (3) do not control for year and industry effects, and both columns (2) and (4) control for year and industry effects. The coefficients of IP and SP in columns (1) and (3) are positive and both are significant at the 1% level of significance, while the coefficients of IP and SP in columns (2) and (4) are both positive and both are significant at the 10% level of significance. This indicates that the involvement of insurers positively contributes to the technological innovation of firms. This may be due to the fact that insurance companies, as important institutional investors in the market, have more positive behavioral motivations and strong information gathering and risk management capabilities, which in turn will influence the investment decisions of companies, especially to invest more in R&D, thus improving the level of technological innovation of companies. Hypothesis H1a is tested.

**Table 3:** Test of the relationship between insurers' intervention and technological innovation.

	**LnTI**			
	**(1)**	**(2)**	**(3)**	**(4)**
IP	0.4013^***^	0.1524^*^		
	(0.0904)	(0.0725)		
SP			33.6115^***^	15.8366^*^
			(8.6476)	(7.7660)
TQ		0.0854^*^		0.0861^*^
		(0.0483)		(0.0483)
CFLOW		−3.6519		−3.6646
		(4.5977)		(4.5974)
SALES		0.1452		0.1468
		(0.1161)		(0.1160)
TOER		−0.0932^*^		−0.0922^*^
		(0.0523)		(0.0523)
LEV		0.3500		0.3427
		(0.4092)		(0.4085)
BTM		0.0053		0.0084
		(0.1635)		(0.1634)
ROA		−2.1316		−2.1569
		(1.6475)		(1.6472)
SIZE		0.6042^***^		0.6060^***^
		(0.0835)		(0.0830)
SOE		−0.0435		−0.0445
		(0.1384)		(0.1383)
SPRT		−0.9317		−0.9403
		(0.7629)		(0.7631)
TAT		0.4361^**^		0.4398^**^
		(0.2036)		(0.2036)
_cons	2.6933^***^	−10.7720^***^	2.7002^***^	−10.8152^***^
	(0.0277)	(1.7343)	(0.0276)	(1.7246)
Year	YES	YES	YES	YES
Indu	YES	YES	YES	YES
R^2^	0.0028	0.0978	0.0021	0.0979
N	3,381	3,381	3,381	3,381

Standard errors are in brackets below the coefficients, and ^*^, ^**^, and ^***^represent significant at the 10, 5, and 1% levels, respectively.

Table 4 reports the test of the relationship between insurers' intervention, separation of two rights and technological innovation with LnTI as the explanatory variable and IP and SP as the explanatory variables, where both column (1) and column (2) control for year and industry effects. The coefficients of IP and SP in columns (1) and (2) are positive and significant at the 1% level of significance, while the coefficients of IP_SPRT (the interaction term between IP and SPRT) and SP_SPRT (the interaction term between SP and SPRT) are negative and significant at the 1% level of significance. This indicates that although the involvement of insurers increases the technological innovation capacity of the firm, the separation of two rights negatively moderates the relationship between it and technological innovation. This is because although insurers have a positive incentive to effectively supervise the company and provide proper guidance in investment decisions to help improve the company's technological innovation capability, but with a high degree of separation of two rights, the company will not have enough capital to invest in R&D. The insurance company will not consider the long-term development goals of the listed company for its own development, on the contrary will collude with the company's management to the company's interests, and the tunnel effect is obvious, which will eventually be detrimental to the company's overall performance growth. At the same time, the R&D investment is reduced and enters a vicious cycle process, and the level of technological innovation naturally declines. Hypothesis H2b is supported.

**Table 4:** Test of the relationship between insurers' intervention, separation of two rights and technological innovation.

	**(1)**	**(2)**
	**LnTI**	**LnTI**
IP	0.2648^*^	
	(0.1486)	
IP_SPRT	−2.0689^*^	
	(1.0388)	
SP		21.8119^*^
		(10.4361)
SP_SPRT		−1.05^*^
		(0.6184)
TQ	0.0848^*^	0.0858^*^
	(0.0482)	(0.0482)
CFLOW	−3.6435	−3.6513
	(4.5905)	(4.5943)
SALES	0.1442	0.1464
	(0.1161)	(0.1161)
TOER	−0.0929^*^	−0.0923^*^
	(0.0523)	(0.0524)
LEV	0.3429	0.3377
	(0.4088)	(0.4082)
BTM	0.0008	0.0067
	(0.1635)	(0.1635)
ROA	−2.1082	−2.1421
	(1.6485)	(1.6484)
SIZE	0.6060^***^	0.6069^***^
	(0.0835)	(0.0830)
SOE	−0.0424	−0.0443
	(0.1384)	(0.1384)
SPRT	−0.6013	−0.7709
	(0.7947)	(0.7931)
TAT	0.4380^**^	0.4421^**^
	(0.2032)	(0.2035)
_cons	−10.8270^***^	−10.8449^***^
	(1.7332)	(1.7244)
Year	YES	YES
Indu	YES	YES
R^2^	0.0984	0.0981
N	3,381	3,381

Standard errors are in brackets below the coefficients, and ^*^, ^**^, and ^***^represent significant at the 10, 5, and 1% levels, respectively.

Table 5 reports the regression results for the sub-sample of SOEs and private firms on the relationship between insurers' intervention, separation of two rights and technological innovation, with LnTI as the explanatory variable and IP and SP as the explanatory variables, where columns (1)–(8) control for year and industry effects. Columns (1)–(4) are for SOEs and columns (5)–(8) are for private firms. The coefficients of IP and SP in columns (1) and (3) are positive but insignificant, the coefficients of IP and SP in columns (2) and (4) are positive and significant at the 5% and 10% levels of significance, respectively, the coefficient of IP_SPRT (interaction term between IP and SPRT) is negative but insignificant, and the coefficient of SP_SPRT (interaction term between SP and SPRT) is negative and significant at the 10% significance level, while the coefficients of IP, SP, IP_SPRT, and SP_SPRT in columns (5)–(8) are insignificant. This indicates that the negative moderating effect of separation of two rights on insurers' intervention and technological innovation is more pronounced in the SOE sample. In SOEs, the agency problem is more severe and more likely to involve insurers in collusive behavior with management's interests, thus engaging in opportunistic speculative behavior, and inefficient investment decisions will inevitably be detrimental to the development of firms' technological innovation capabilities. Further support is provided for hypothesis H3.

**Table 5:** Regression results for the sub–sample of SOEs and private enterprises.

	**LnTI**							
	**SOE** = **1**				**SOE** = **0**			
	**(1)**	**(2)**	**(3)**	**(4)**	**(5)**	**(6)**	**(7)**	**(8)**
IP	0.2800	0.4198^**^			0.0041	−0.0018		
	(0.1754)	(0.2003)			(0.1700)	(0.2209)		
IP_SPRT		−3.4857				0.0872		
		(2.1284)				(2.1093)		
SP			22.3000	32.6903^*^			8.0156	5.6236
			(17.3113)	(19.8268)			(15.9976)	(20.9657)
SP_SPRT				−2.52^*^				34.4754
				(1.2711)				(191.7803)
TQ	0.0706	0.0656	0.0725	0.0688	0.0579	0.0578	0.0584	0.0581
	(0.0938)	(0.0933)	(0.0938)	(0.0932)	(0.0591)	(0.0592)	(0.0592)	(0.0592)
CFLOW	2.0212	2.3385	2.1134	2.3678	−8.6211	−8.6155	−8.6178	−8.5953
	(6.9702)	(6.9670)	(6.9673)	(6.9775)	(6.0597)	(6.0709)	(6.0554)	(6.0668)
SALES	0.1563	0.1596	0.1582	0.1607	0.1236	0.1237	0.1252	0.1255
	(0.1831)	(0.1820)	(0.1827)	(0.1823)	(0.1502)	(0.1502)	(0.1504)	(0.1504)
TOER	−0.0740	−0.0740	−0.0728	−0.0735	−0.1252^**^	−0.1253^**^	−0.1224^*^	−0.1226^*^
	(0.0967)	(0.0969)	(0.0967)	(0.0967)	(0.0626)	(0.0627)	(0.0627)	(0.0627)
LEV	−0.4535	−0.4749	−0.4644	−0.4840	1.2721^**^	1.2725^**^	1.2754^**^	1.2773^**^
	(0.7078)	(0.7027)	(0.7072)	(0.7025)	(0.5186)	(0.5188)	(0.5170)	(0.5169)
BTM	0.1011	0.0928	0.1030	0.0989	−0.3476	−0.3477	−0.3430	−0.3431
	(0.2199)	(0.2201)	(0.2198)	(0.2201)	(0.2454)	(0.2454)	(0.2451)	(0.2450)
ROA	−1.3549	−1.2967	−1.3532	−1.2980	−2.5354	−2.5350	−2.5576	−2.5571
	(2.6419)	(2.6445)	(2.6427)	(2.6447)	(2.1050)	(2.1051)	(2.1055)	(2.1056)
SIZE	0.7854^***^	0.7867^***^	0.7948^***^	0.7962^***^	0.4254^***^	0.4252^***^	0.4218^***^	0.4212^***^
	(0.1246)	(0.1243)	(0.1234)	(0.1231)	(0.1178)	(0.1184)	(0.1172)	(0.1176)
SPRT	−1.1713	−0.5152	−1.1803	−0.7289	−0.7153	−0.7274	−0.7227	−0.7732
	(1.3013)	(1.3213)	(1.3011)	(1.3162)	(0.9402)	(0.9921)	(0.9399)	(0.9880)
TAT	0.4195	0.4106	0.4228	0.4203	0.4987^*^	0.4983^*^	0.5038^*^	0.5019^*^
	(0.3051)	(0.3038)	(0.3059)	(0.3054)	(0.2676)	(0.2675)	(0.2677)	(0.2674)
_cons	−14.7701^***^	−14.8133^***^	−14.9834^***^	−15.0262^***^	−6.8773^***^	−6.8721^***^	−6.8211^***^	−6.8031^***^
	(2.7268)	(2.7189)	(2.7003)	(2.6942)	(2.3808)	(2.3956)	(2.3722)	(2.3819)
Year	YES	YES	YES	YES	YES	YES	YES	YES
Indu	YES	YES	YES	YES	YES	YES	YES	YES
R^2^	0.1578	0.1594	0.1572	0.1581	0.0716	0.0716	0.0718	0.0718
N	1,361	1,361	1,361	1,361	2,020	2,020	2,020	2,020

Standard errors are in brackets below the coefficients, and ^*^, ^**^, and ^***^represent significant at the 10, 5, and 1% levels, respectively.

### 4.3. Robustness tests

In order to prevent endogeneity problems caused by sample selection bias, this paper further adopts the PSM method to select paired samples of companies by the propensity score (PS), and conducts radius matching, kernel matching and nearest neighbor matching respectively to test the relationship between “insurers' intervention, separation of two rights and firm's technological innovation”. Eight firm characteristics variables, such as book-to-market ratio (BTM), free cash flow (CFLOW), asset-to-liability ratio (LEV), return on assets (ROA), sales ratio (SALES), asset size (SIZE), Tobin's Q (TQ), and turnover ratio (TOER), were selected to test the relationship between “insurers' intervention, separation of two rights, and firm technological innovation” to build propensity models. Logit models were used to estimate the propensity score value (PS value) for each company's two power separation and to test the differences of the dependent variables between the two sample groups. Table 6 shows the results of the PSM test, and the t-value tests for the pre- and post-matching samples are not significant and the differences are small for either matching method. Further support is provided for hypothesis 1 and hypothesis H2a.

**Table 6:** PSM regression results.

	**LnTI**					
	**Radius matching**		**Kernel matching**		**Nearest neighbor matching**	
	**(1)**	**(2)**	**(3)**	**(4)**	**(5)**	**(6)**
IP	0.2807^*^		0.2807^*^		0.2807^*^	
	(−0.147)		(−0.148)		(−0.148)	
IP_SPRT	−2.4971^*^		−2.4972^*^		−2.4970^*^	
	(−1.3292)		(−1.3293)		(−1.3291)	
SP		23.1943^*^		23.1943^*^		23.1942^*^
		(−13.3619)		(−13.3619)		(−13.3617)
SP_SPRT		−1.52^*^		−1.52^*^		−1.51^*^
		(−0.7513)		(−0.7513)		(−0.7512)
TQ	0.0789	0.0805^*^	0.0789	0.0805^*^	0.0789	0.0805^*^
	(−0.0485)	(−0.0487)	(−0.0483)	(−0.0487)	(−0.0486)	(−0.0487)
CFLOW	−2.2302	−2.3154	−2.2302	−2.3154	−2.2302	−2.3154
	(−4.4738)	(−4.4791)	(−4.4738)	(−4.4791)	(−4.4738)	(−4.4791)
SALES	0.0871	0.0886	0.0871	0.0886	0.0871	0.0886
	(−0.1142)	(−0.1145)	(−0.1143)	(−0.1145)	(−0.1145)	(−0.1145)
TOER	−0.0971^*^	−0.0957^*^	−0.0971^*^	−0.0957^*^	−0.0971^*^	−0.0957^*^
	(−0.0524)	(−0.0525)	(−0.0524)	(−0.0525)	(−0.0524)	(−0.0525)
LEV	0.5314	0.522	0.5314	0.522	0.5314	0.522
	(−0.3926)	(−0.3923)	(−0.3928)	(−0.3923)	(−0.3927)	(−0.3923)
BTM	0.005	0.006	0.005	0.006	0.005	0.006
	(−0.1651)	(−0.1653)	(−0.1651)	(−0.1653)	(−0.1651)	(−0.1653)
ROA	−1.0455	−1.0787	−1.0455	−1.0787	−1.0455	−1.0787
	(−1.5857)	(−1.5848)	(−1.5856)	(−1.5848)	(−1.5858)	(−1.5848)
SIZE	0.5893^***^	0.5905^***^	0.5893^***^	0.5905^***^	0.5893^***^	0.5905^***^
	(−0.082)	(−0.0825)	(−0.085)	(−0.0825)	(−0.083)	(−0.0825)
SOE	0.0082	0.0124	0.0082	0.0124	0.0082	0.0124
	(−0.1344)	(−0.1344)	(−0.1344)	(−0.1344)	(−0.1344)	(−0.1344)
_cons	−10.3784^***^	−10.4110^***^	−10.3784^***^	−10.4110^***^	−10.3784^***^	−10.4110^***^
	(−1.7125)	(−1.7046)	(−1.7125)	(−1.7046)	(−1.7125)	(−1.7046)
Year	YES	YES	YES	YES	YES	YES
Indu	YES	YES	YES	YES	YES	YES
R^2^	0.0955	0.0950	0.0954	0.0951	0.0953	0.0951
N	3381	3381	3381	3381	3381	3381

Standard errors are in brackets below the coefficients, and^*^, ^**^, and ^***^represent significant at the 10, 5, and 1% levels, respectively.

Meanwhile, since the separation of two rights has an important relationship with the equity structure, the equity concentration indicator (the first largest shareholder's shareholding) is selected instead of SPRT for the robustness test, and Table 7 is the result of the robustness test, which is consistent with the results of the previous study.

**Table 7:** Robustness tests.

	**LnTI**			
	**(1)**	**(2)**	**(3)**	**(4)**
IP	0.1524^*^	0.0682^*^		
	(0.0825)	(0.0367)		
IP_TOPHLD		−0.5742^*^		
		(0.2478)		
SP			15.8366^*^	6.1695^*^
			(8.7660)	(3.6793)
SP_TOPHLD				−26.1016^*^
				(15.7961)
TQ	0.0854^*^	0.0826^*^	0.0861^*^	0.0848^*^
	(0.0483)	(0.0479)	(0.0483)	(0.0479)
CFLOW	−3.6519	−3.5891	−3.6646	−3.6243
	(4.5977)	(4.5989)	(4.5974)	(4.5989)
SALES	0.1452	0.1430	0.1468	0.1463
	(0.1161)	(0.1163)	(0.1160)	(0.1162)
TOER	−0.0932^*^	−0.0923^*^	−0.0922^*^	−0.0918^*^
	(0.0523)	(0.0521)	(0.0523)	(0.0522)
LEV	0.3500	0.3550	0.3427	0.3436
	(0.4092)	(0.4091)	(0.4085)	(0.4085)
BTM	0.0053	0.0022	0.0084	0.0075
	(0.1635)	(0.1632)	(0.1634)	(0.1634)
ROA	−2.1316	−2.1182	−2.1569	−2.1543
	(1.6475)	(1.6474)	(1.6472)	(1.6471)
SIZE	0.6042^***^	0.6009^***^	0.6060^***^	0.6048^***^
	(0.0835)	(0.0837)	(0.0830)	(0.0831)
SOE	−0.0435	−0.0476	−0.0445	−0.0469
	(0.1384)	(0.1381)	(0.1383)	(0.1378)
TAT	0.4361^**^	0.4312^**^	0.4398^**^	0.4379^**^
	(0.2036)	(0.2038)	(0.2036)	(0.2040)
_cons	−10.7720^***^	−10.6929^***^	−10.8152^***^	−10.7858^***^
	(1.7343)	(1.7379)	(1.7246)	(1.7269)
Year	YES	YES	YES	YES
Indu	YES	YES	YES	YES
R^2^	0.0978	0.0980	0.0979	0.0980
N	3,381	3,381	3,381	3,381

Standard errors are in brackets below the coefficients, and ^*^, ^**^, and ^***^represent significant at the 10, 5, and 1% levels, respectively.

## 5. Conclusion and policy recommendations

Technological innovation as a non-investment activity of enterprises, listed companies have certain advantages. But this advantage is likely to be weakened by the agency conflict of separation of two rights, and in the context of capital market innovation as a hot spot, it is an inevitable requirement for companies to improve their technological innovation capability as a result of the development of internal and external environment. As an important institutional investor in the market, how will the involvement of insurance companies affect technological innovation? What is the effect of the separation of two rights of the company on their relationship? This paper analyzes the relationship between insurers' intervention, separation of two rights and technological innovation using the data of listed companies from 2007 to 2018 as the research sample. The results show that insurers' intervention has a positive relationship with the technological innovation output of companies, and has a positive mediating effect on technological innovation by affecting agency costs, and this effect is more obvious in the sample of state-owned enterprises. This is mainly because the participation of insurance companies in listed companies will have a positive effect on technological innovation through the supervision and management of the companies, thus affecting their performance improvement and increasing their R&D investment; however, due to the serious agency conflict problem in Chinese listed companies, the management will involve the institutional investors in their collusion group and will not focus on the long-term development and strategic investment of the companies, which will be detrimental to the development of technological. The findings of this paper are useful for strengthening the internal equity of companies, and have profound implications for strengthening the internal equity structure of companies and state regulation of the capital market.

In the end, this paper proposes the following recommendations: (1) as an emerging capital market, China's various operation mechanisms are still unsound, and no effective restraint mechanism has been formed for listed companies, so effective external supervision should be carried out for the loopholes in the development of listed companies, especially the agency conflict of separation of two rights; (2) the internal governance structure of listed companies should be improved to form relatively effective institutional constraints, and an effective governance system should be formed for agency conflicts, especially the behavior of controlling shareholders should be strictly restrained to prevent their “emptying” of the company's internal interests; (3) for institutional investors such as insurance companies, the country should provide effective policy guidance and constraints to avoid collusion of interests between insurance companies and company management, so as to ensure effective corporate governance and investment management, avoiding the waste of financial resources and improving the efficiency of capital allocation.

## Data availability statement

The original contributions presented in the study are included in the article/supplementary material, further inquiries can be directed to the corresponding author.

## Author contributions

BX: conceptualization, writing-review, and editing. FH: conceptualization, methodology, and writing-original draft. All authors contributed to the article and approved the submitted version.

## References

[1] Hong M Zhang T Wang G. Heterogeneous Institutional Investors and Enterprises' Technology Innovation: An Empirical Test Based on Institutional Investors in Different Terms. Forum on Science and Technology in China. (2018), p. 57–70.

[2] Hall BH Lerner J. The financing of RandD and innovation. Handbook Econ Innovat. (2010) 1:609–39. 10.1016/S0169-7218(10)01014-236301388

[3] Borochin P Yang J. The effects of institutional investor objectives on firm valuation and governance. J Financ Econ. (2017) 126:171–199. 10.1016/j.jfineco.2017.06.013

[4] Bushee BJ. The influence of institutional investors on myopic RandD investment behavior. Account Rev. (1998) 73:305–333.

[5] Ahmed D Shahab Y Ullah F Ye Z. Investor sentiment and insurers' financial stability: do sovereign rating matter? Geneva Papers Risk Insurance Issues Practice. (2019) 45:281–312. 10.1057/s41288-020-00160-z

[6] Mihaela B Ovidiu S. Herding behavior of institutional investors in Romania. Empirical Anal Rev Econ Business Stud. (2017) 10:115–30. 10.1515/rebs-2017-0057

[7] Eng LL Shackell M. The implications of long-term performance plans and institutional ownership for firms' RandD investments. J Account Audit Financ. (2011) 16:117–39. 10.1177/0148558X0101600204

[8] Aghion P Van Reenen J Zingales L. Innovation and institutional ownership. Am Econ Rev. (2013) 103:277–304. 10.1257/aer.103.1.277

[9] Zhao H Xia H. An empirical study on relationship between the stock holdings of institutional investors and companies' innovation activity. China Soft Sci. (2009) 5:33–54. 10.3969/j.issn.1002-9753.2009.05.005

[10] Bo X Wu L. The governance roles of state-owned controlling and institutional investors perspective a of earnings manage. Econ Res J. (2009) 44:81–91.

[11] Gui A Haron H Sabrina C Timoti J Yusuf M Pratiwi SD. Information technology risk management using frap (a case of insurance company). Appl Mech Mater. (2013) 437:857–60. 10.4028/www.scientific.net/AMM.437.857

[12] Bhatnagar V Ranjan J Singh R. Analytical customer relationship management in insurance industry using data mining: a case study of Indian insurance company. Int J Network Virtual Organiz. (2011) 9:331. 10.1504/IJNVO.2011.043803

[13] Mcnulty T Nordberg D. Ownership, activism and engagement: institutional investors as active owners. Corporate Governance Int Rev. (2016) 24:346–58. 10.1111/corg.12143

[14] Hu S Jin L Liu H. Empirical analysis of the impact of financial structure on technological innovation. Forum Sci Technol China. (2019) 10:33–42.

[15] Ahmed D Xie Y Issam K. Investor confidence and life insurance demand: can economic condition limit life insurance business? Int J Emerg Markets. (2021) 1746–8809. 10.1108/IJOEM-06-2020-0650

[16] Zamri A Haslindar I Jasman T. Institutional investor behavioral biases: syntheses of theory and evidence. Manage Res Rev. (2017) 40:578–603. 10.1108/MRR-04-2016-0091

[17] Pucheta-Martínez MC López-Zamora B. Engagement of directors representing institutional investors on environmental disclosure. Corporate Soc Responsibil Environ Manage. (2018) 25:1108–20. 10.1002/csr.1525

[18] Polonchek J Miller RK. The impact of management communications on insurance company share repurchases. J Insurance Issues. (2005) 28:183–210.

[19] Porta RL Lopez-De-Silanes F Shleifer A. Corporate ownership around the world. J Finance. (1999) 54:471–517. 10.1111/0022-1082.00115

[20] Xiong Y. Private pyramid structure, product market competition and innovation investment. Soft Sci. (2014) 28:17–20. 10.3969/j.issn.1001-8409.2014.08.004

[21] Bebchuk LA Cohen A Hirst S. The agency problems of institutional investors. Soc Sci Electron Publ. (2017) 31:89–112. 10.1257/jep.31.3.89

[22] Xu P Bai G. Research on the relationship between institutional environment and enterprise technological innovation from the perspective of dynamic competition technological innovation—empirical evidence from listed companies within the framework of enterprise groups. Finance Econ. (2019) 10:94–105.

[23] Liu Y Sheng Y. Empirical study on the relationship between control-ownership divergence and corporate technological innovation: moderating effect of property rights and deviation of firm performance effect. Sci Decis Making. (2018) 60–82. 10.3773/j.issn.1006-4885.2018.03.060

[24] Chen J Tang X Zhao H Jin C. Difference on ultimate ownership structure, the degree of divergence in control and ownership, and corporate innovation. J Shanxi Finance Econ Univ. (2013) 35:81–91.

[25] Xu X Tang Y. An analysis of inhibitory effect of pyramid structure over innovatively technological performance—an empirical research based on the data of small and medium-sized listed companies in China. Theory J. (2013) 3:62–6. 10.3969/j.issn.1002-3909.2013.03.013

[26] Chang X Fu K Low A Zhang W. Non-executive employee stock options and corporate innovation. J Financial Econ. (2015) 115, 168–188. 10.1016/j.jfineco.2014.09.002

